# Factors associated with *Leptospira* serodiagnosis in febrile patients at public health centers in Makassar, Indonesia: a cross-sectional study

**DOI:** 10.11604/pamj.2024.49.113.45645

**Published:** 2024-12-09

**Authors:** Cheria Cahyaningtyas, Lisa Tenriesa Muslich, Baedah Madjid, Andi Rofian Sultan, Firdaus Hamid, Mochammad Hatta

**Affiliations:** 1 Department of Clinical Microbiology, Faculty of Medicine, Hasanuddin University, Makassar, Indonesia,; 2 Microbiology Laboratory, Universitas Hasanuddin Hospital, Makassar, Indonesia

**Keywords:** Leptospirosis, Leptospira, IgM, ELISA, rapid diagnostics

## Abstract

**Introduction:**

leptospirosis is a globally prevalent zoonotic disease that can lead to outbreaks with significant public health implications. In Indonesia, particularly in East Java Province and South Sulawesi, reported cases of leptospirosis have been increasing. Diagnosis typically relies on the Leptospira rapid test and ELISA. This study aimed to assess the association between high-risk populations and Leptospira infection.

**Methods:**

this cross-sectional observational study included febrile patients hospitalized at Public Health Centers in Makassar City. Blood samples were collected from eligible participants and tested using both the IgM ELISA and Standard Q Leptospira IgM/IgG rapid test.

**Results:**

of the 78 participants, 51% were female, and 64% lived in high-risk areas. The Standard Q Leptospira IgM/IgG test identified 1.3% as positive, while the IgM ELISA detected 2.6% positive cases. The two diagnostic methods showed strong concordance (88.7%). No significant differences were found between infection rates and factors such as age, gender, occupation, or lifestyle.

**Conclusion:**

the incidence of leptospirosis in Makassar was low. There was good agreement between the rapid test and ELISA tests. No significant association was observed between Leptospira infection and variables such as age, gender, occupation, or lifestyle.

## Introduction

Leptospirosis is a globally widespread zoonotic disease that can cause outbreaks with significant public health implications [1-3]. Leptospirosis is caused by pathogenic spirochetes from the genus *Leptospira*, with over 200 serovars, primarily due to *Leptospira interrogans* and *Leptospira kirschneri*. Leptospirosis represents a significant yet often overlooked bacterial infection that is endemic in subtropical and tropical regions. Although its identified prevalence is minimal compared to other tropical diseases within Indonesia, particularly dengue fever, leptospirosis continues to pose a considerable public health challenge, particularly in areas susceptible to substantial flooding and high rainfall [3]. The burden of leptospirosis in Indonesia is significant, with the country ranking third globally in terms of case fatality rate (CFR) and experiencing widespread incidence across various regions [4]. In 2021, 734 cases of leptospirosis were reported in Indonesia, resulting in 84 deaths across eight provinces, with the majority occurring in East Java Province [5,6]. In the year 2020, the Indonesian Ministry of Health documented a total of 1,170 cases of leptospirosis, which resulted in 106 fatalities, yielding a CFR of 9.1% across eight provinces. The provinces that reported incidences of leptospirosis encompassed DKI Jakarta, West Java, Central Java, DI Yogyakarta, East Java, Maluku, South Sulawesi, and North Kalimantan [7].

Clinicians in Indonesia are predominantly unaware of the clinical features associated with leptospirosis, which mainly involve non-specific anicteric and influenza-like symptoms in many cases. Accompanying symptoms such as myalgia, chills, cephalalgia, and acute fever overlap with those associated with dengue and typhoid fevers [8], which are more prevalent than leptospirosis within the Indonesian context. The prompt diagnosis of this treatable bacterial infection is imperative for effectively managing cases. Instances that remain untreated are subjected to an elevated risk of advancing to the severe form of acute kidney injury due to Weil´s disease, which is characterized by a case fatality rate exceeding 70% [3,9]. The serological assay is regarded as the 'gold standard' of the microscopic agglutination test (MAT), which necessitates specialized personnel and substantial resources, is restricted to three facilities throughout Indonesia [3]. Alternative diagnostic methods, including the *Leptospira* rapid test and ELISA, which possess the capability to identify infections in the early stages of the disease, are frequently available in resource-constrained environments where leptospirosis is prevalent. The *Leptospira* rapid test is used for the detection of IgM/IgG antibodies to *Leptospira* bacteria in human plasma, serum, or whole blood specimens, enabling rapid screening and diagnosis of suspected leptospirosis cases [10,11]. ELISA is another diagnostic tool used to detect *Leptospira*-specific IgM and IgG antibodies in the sera of patients infected with various *Leptospira* serovars [12]. Aims and objectives: this study focuses on the incidence of leptospirosis in humans, particularly in Makassar City, with the aim of increasing awareness among researchers about this disease, especially in individuals at high risk of asymptomatic *Leptospira* infection. The objective of the study is to determine the incidence of *Leptospira* cases and to identify risk factors for *Leptospira* infection in patients presenting with fever symptoms in Makassar City, utilizing both ELISA and rapid test methods.

## Methods

**Study design:** this descriptive observational study employed a cross-sectional approach.

**Study setting:** this study took place at Kassi-Kassi, Patingaloa, Antang, and Batua public health centers in Makassar City, Indonesia. The study period extended from September 2023 to May 2024.

**Participants:** all febrile patients were admitted to four Public Health Centers.

**Study size:** the sample size was determined using the Slovin formula [13], where N (the number of febrile patients in four Public Health Centers) and e (absolute precision is 10%) obtained a minimum number of participants of 68.

**Inclusion and exclusion criteria:** the inclusion criteria were febrile patients (body temperature above 37.5°C), aged between 5 and 65 years. The exclusion criteria included patients diagnosed with other infections and patients with contaminated or damaged blood samples.

**High-risk occupation:** the study includes individuals employed as janitors, sewer maintenance workers, slaughterhouse workers, scavengers, and pet shop workers.

**High-risk lifestyle:** participants in the study who regularly play/work in the soil without wearing footwear, swim in rivers or wash in rivers, and live-in flood-prone areas.

**Data source:** demographic data was collected via medical records, which included sex and age. Blood samples were drawn from each participant via venipuncture using a 2-3mL syringe with a 21 G needle and collected into sterile 3mL vacutainer blood tubes. The blood samples were stored in a cool box and later refrigerated at -20 °C. The serum was analyzed using two methods: i) ELISA for the detection of human IgM, performed with the Human IgM-ELISA kit from Virion\Serion GmbH (Wurzburg, Germany, catalog number ESR125M) following the manufacturer's protocol, and; ii) a rapid test for *Leptospira* IgM/IgG antibodies using the SD Biosensor kit (Chungcheongbuk-do, Republic of Korea, catalog number 09LEP10D).

**Statistical methods:** data was processed using Microsoft Excel and analyzed with SPSS version 26 (Armonk, NY: IBM Corp.). Fisher's exact test was used to evaluate the correlation between risk factors and *Leptospira* infection, with a significance threshold of p ≤ 0.05.

**Potential bias:** all assays (ELISA and rapid test) were performed in duplicate according to each manufacturer's protocol.

**Ethical considerations:** ethical approval was obtained from the Institutional Review Board of the Faculty of Medicine, Hasanuddin University (approval number: UH23080545, issued on September 4, 2023). All procedures followed the principles of the 1964 Declaration of Helsinki and its later amendments, Good Clinical Practice guidelines, and the International Conference on Harmonization standards

## Results

**Participants:** in this study, the 78 participants were nearly equally distributed between genders, with 38 male participants (48.7%) and 40 female participants (51.3%).

**Descriptive data:** the largest age group was 12-25 years, accounting for 38 participants (48.7%), while no participants were in the 0-5 years age range. In addition, 50 participants (64.1%) resided in high-risk areas, such as flood-prone regions or areas near landfills, 6 participants (7.7%) had occupations with an increased risk of exposure to *Leptospira*, and 14 participants (17.9%) had lifestyles that posed a risk for exposure (Table 1).

**Table 1:** demographic characteristics of 78 Febrile patients at public health centers in Makassar, Indonesia, from September 2023 to May 2024

Variables	n (%)
**Sex**	
Male	38 (48.7)
Female	40 (51.3)
**Age (years)**	
0-5	0 (0)
6-11	11 (14.1)
12-25	38 (48.7)
26-45	22 (28.2)
46-65	7 (8.9)
**Risk factors**	
High-risk occupation	6 (7.7)
Residing in flooded area/near landfill	3 (3.9)
High-risk lifestyle	14 (17.9)
**Primary healthcare facility**	
Batua	18 (23.1)
Antang Perumnas	77 (98.7)
Kassi-Kassi	5 (6.4)
Patingalloa	3 (3.9)
**Standard Q *Leptospira* IgM/IgG test**	
Positive	1 (1.3)
Negative	77 (98.7)
***Leptospira* IgM ELISA**	
Positive	2 (2.6)
Negative	76 (97.4)

**Outcome data:** based on the Standard Q *Leptospira* IgM/IgG test, only 1 participant (1.3%) tested positive, while the *Leptospira* IgM ELISA test identified 2 participants (2.6%) as positive.

**Main results:** the study found that most samples in both the child to the late adolescent group and the early to late adult group tested negative for *Leptospira* IgM using ELISA and Standard Q *Leptospira* IgM/IgG test. There was no significant difference in the incidence of leptospirosis across age groups, as indicated by the statistical analysis (Table 2, p>0.05). Therefore, the results suggest that age is not associated with the incidence of leptospirosis. Most male and female participants tested negative for *Leptospira* IgM using ELISA and Standard Q *Leptospira* IgM/IgG test. There was no statistically significant difference in the proportion of positive and negative results between male and female groups for either test. Overall, the analysis showed no association between sex and the incidence of leptospirosis. No participants with high-risk occupations tested positive for leptospirosis using either the *Leptospira* IgM ELISA or the Standard Q *Leptospira* IgM/IgG test. There was no significant association between high-risk occupations and the incidence of leptospirosis (Table 2). In Table 2 presents the results of *Leptospira* IgM ELISA and Standard Q *Leptospira* IgM/IgG tests. Among high-risk lifestyle participants, 7.1% tested positive for *Leptospira* in both tests, while 2.1% of low-risk lifestyle participants tested positive. However, there was no significant difference between the two groups, suggesting no statistical association between high-risk lifestyles and the incidence of leptospirosis based on both tests (p > 0.05).

**Table 2:** correlation between age, sex, occupation, lifestyle, and *Leptospira* infection among febrile patients at public health centers in Makassar, Indonesia, from September 2023 to May 2024

Variable	*Leptospira* IgM ELISA			Standard Q *Leptospira* IgM/IgG		
	Negative (%)	Positive (%)	p-value†	Negative (%)	Positive (%)	p-value†
**Age groups**						
Child to Late Adolescent (6-25 years)	47 (96)	2 (4)	1	48 (98)	1 (2)	1
Early Adult to Late adult (26-65 years)	29 (100)	0		29 (100)	0	
**Sex**						
Male	37 (97.4)	1 (2.6)	1	37 (97.4)	1 (2.6)	0.488
Female	39 (97.5)	1 (2.5)		40 (100)	0	
**Occupation**						
High risk	6 (100)	0	1	6 (100)	0	1
Low risk	53 (96.4)	2 (3.6)		54 (98.2)	1 (1.8)	
**Lifestyle**						
High risk	13 (92.9)	1 (7.1)	0.409	13 (92.9)	1 (7.1)	0.23
Low risk	46 (97.9)	1 (2.1)		47 (100)	0	

**†:** Fisher exact test

**Other analyses:** a comparison of the Standard Q *Leptospira* IgM/IgG Test results with the IgM ELISA results showed good agreement between the two methods in participants suspected of having leptospirosis (Table 3). The observed concordance rate was 88.7%, with a Cohen's kappa value of 0.66, indicating substantial agreement (kappa 0.61-0.80). One serum sample tested positive by both the Standard Q *Leptospira* IgM/IgG Test and IgM ELISA tests, and 76 serum samples tested negative by both methods. One serum sample tested positive by IgM ELISA but negative by the Standard Q *Leptospira* IgM/IgG Test. No sample was negative by IgM ELISA but positive by the Standard Q *Leptospira* IgM/IgG Test.

**Table 3:** comparison of the *Leptospira* IgM ELISA and Standard Q *Leptospira* IgM/IgG methods among febrile patients at public health centers in Makassar, Indonesia, from September 2023 to May 2024

Variables		Standard Q *Leptospira* IgM/IgG		Observed suitability	Cohen's Kappa
		Negative (%)	Positive (%)		
*Leptospira* IgM ELISA	Positive (%)	1 (1.3)	1 (1.3)	88.7%	0.66
	Negative (%)	76 (97.4)	0		

## Discussion

The prevalence of leptospirosis in this study found no significant association between leptospirosis incidence and factors such as age, gender, or lifestyle. This study showed that the percentage of leptospirosis cases in Makassar was low. In 2022, 1,408 cases of leptospirosis were reported in Indonesia, with a case fatality rate of 9.87% (139 deaths). This figure represents an increase compared to 2019-2021. An outbreak was reported in Pangkep District, South Sulawesi, in March 2023, with 4 cases and one fatality [14]. In this study, of the 78 febrile participants recruited from several health centers in Makassar, 2 samples (2.6%) tested positive for leptospirosis using the IgM ELISA method, while only 1 sample (1.3%) tested positive using the Standard Q *Leptospira* IgM/IgG Test. These results are lower than those of a previous study conducted in the same city, where 5.96% (24 cases) were identified as positive by the microscopic agglutination test (MAT) and IgM ELISA, and 5.5% (22 cases) by the IgM Dipstick method [15]. In another study conducted in seven Indonesian cities from 2013 to 2016, 3.1% of cases (45 cases) were confirmed, while 0.4% of cases were classified as probable leptospirosis [3]. In this study, 1 serum sample tested positive with both the Standard Q *Leptospira* IgM/IgG Test and IgM ELISA, and 76 samples were negative in both tests. One sample tested positive with IgM ELISA but negative with the Standard Q *Leptospira* IgM/IgG Test, and one sample was positive by the Standard Q *Leptospira* IgM/IgG Test but negative by IgM ELISA. The comparison of Standard Q *Leptospira*, IgM/IgG Test results with IgM ELISA showed good agreement (88.7%, Kappa value = 0.66) between the two methods in participants suspected of having leptospirosis. These findings are consistent with previous studies, which reported Kappa values between 0.69 and 0.76, indicating a high level of concordance between the dipstick test and IgM ELISA [11]. The dipstick test detected *Leptospira*-specific IgM antibodies in 68.6% of patients with clinical evidence of leptospirosis, whereas IgM ELISA detected such antibodies in 84% of patients with acute *Leptospira* infection [15,16].

The sensitivity of the IgM ELISA for diagnosing acute *Leptospira* infection has been reported to be 84%, with a specificity of 91% [17]. The Leptospirosis IgM/IgG Dipstick test demonstrated a sensitivity of 91.6% and a specificity of 93.3% for detecting *Leptospira*-specific IgM antibodies [15]. Smits *et al*. reported the dipstick test, when used to detect various *Leptospira* serogroups, had a sensitivity of 60.1% for acute-phase serum samples, increasing to 87.4% for convalescent-phase samples [16]. Their study also compared the dipstick test with IgM ELISA and found that the dipstick test detected *Leptospira*-specific IgM antibodies in 87.4% of laboratory-confirmed leptospirosis cases, while the IgM ELISA detected antibodies in 84% of these patients [16]. Overall, the concordance between *Leptospira* IgM ELISA and the *Leptospira* Dipstick IgM/IgG test for the diagnosis of leptospirosis was high, indicating that both methods are effective for detecting *Leptospira*-specific IgM antibodies. In this study, no association was found between age group, gender, or occupational risk and the incidence of leptospirosis, as identified by either Standard Q *Leptospira* IgM/IgG Test or the *Leptospira* IgM ELISA method. However, a previous systematic review in Indonesia reported that leptospirosis was most prevalent among adults aged 20-50 years, males, and individuals working as farmers [18]. Several previous studies have reported a significant association between gender and leptospirosis infection, with males consistently shown at higher risk than females globally. Males are more susceptible to leptospirosis, with higher incidence rates observed in various studies [19,20]. For instance, a study in Albania found a male-to-female ratio of 9: 1 in leptospirosis cases [20], while a study in Malaysia reported that 87% of patients hospitalized for leptospirosis were male [21]. This higher incidence in males is often attributed to increased occupational exposure, as males are more likely to engage in farming, fishing, and automotive repair-activities associated with greater *Leptospira* exposure [20,21].

This higher incidence in males is often attributed to increased occupational exposure, as males are more likely to engage in farming, fishing, and automotive repair-activities associated with greater *Leptospira* exposure [20,21]. However, this explanation may not fully capture the complexity of the relationship between gender and leptospirosis risk [18,19]. In addition, previous studies have shown that males tend to experience more severe outcomes and higher mortality rates than females, despite similar exposure risks. A study in Germany reported a mortality rate of 5% in male patients compared to 1% in female patients [19]. Biological differences, including genetic, immunological, and hormonal factors, may also contribute to these sex differences in leptospirosis risk and severity [19,20]. The strengths of this study lie in its robust design, the use of two diagnostic methods, a focus on high-risk populations, and its contribution to local public health knowledge, all of which enhance its relevance and applicability. Clinically, these findings may help improve clinical diagnostics of leptospirosis by demonstrating that the rapid test serves as a quick and useful tool for field use, while the IgM ELISA provides further confirmation in a laboratory setting. The combination of these two tests could expedite diagnosis and improve patient management, particularly in resource-limited settings.

**Limitations:** limitations of the study include small number of positive cases and its focus on only four community health centers, which may exclude leptospirosis patients who sought care at private clinics or hospitals. Additionally, the literature on *Leptospira* cases diagnosed using both ELISA and rapid test methods remains limited. Furthermore, this study was not conducted during the peak of the rainy season, when flooding-a key risk factor for leptospirosis-is more common.

## Conclusion

This preliminary research found no significant association between leptospirosis incidence and factors such as age, gender, or lifestyle, and the percentage of leptospirosis cases in Makassar probably was low. Further investigation with qPCR and MAT tests, and involving a larger and more diverse participant pool, encompassing all public health centers in Makassar City, Indonesia, is warranted. This would help capture a more comprehensive understanding of leptospirosis prevalence and risk factors in Makassar City, Indonesia. There was good agreement between the Standard Q *Leptospira* IgM/IgG Test and ELISA results, indicating that the rapid test is a simple and effective tool that can be used without specialized equipment.

### What is known about this topic

- Leptospirosis is a globally widespread zoonotic disease that can cause outbreaks with significant public health implications;
- The Leptospira rapid test detects IgM/IgG antibodies enabling rapid screening and diagnosis of suspected leptospirosis cases;
- ELISA is another diagnostic tool used to detect Leptospira-specific IgM and IgG antibodies in the sera of patients infected with various Leptospira serovars.

### What this study adds

- The percentage of leptospirosis cases in Makassar was low;
- There was good agreement between the Standard Q Leptospira IgM/IgG Test and ELISA results, indicating that the rapid test is a simple and effective tool that can be used without specialized equipment.
